# Expression analysis of miRNAs and their putative target genes confirm a preponderant role of transcription factors in the early response of oil palm plants to salinity stress

**DOI:** 10.1186/s12870-021-03296-9

**Published:** 2021-11-08

**Authors:** Fernanda Ferreira Salgado, Letícia Rios Vieira, Vivianny Nayse Belo Silva, André Pereira Leão, Priscila Grynberg, Marcos Mota do Carmo Costa, Roberto Coiti Togawa, Carlos Antônio Ferreira de Sousa, Manoel Teixeira Souza Júnior

**Affiliations:** 1 PGBV - Universidade Federal de Lavras – UFLA, CEP 37200-000, Lavras, MG Brazil; 2 Embrapa Agroenergia, CEP 70770-901, Brasília, DF Brazil; 3 Embrapa Recursos Genéticos e Biotecnologia, CEP 70770-917, Brasília, DF Brazil; 4 Embrapa Meio Norte, Av. Duque de Caxias, 5650, CEP 64.006-245, Teresina, PI Brazil

**Keywords:** Abiotic stress, *Elaeis guineensis*, Transcription factor, Transcriptome, Non-coding RNA

## Abstract

**Background:**

Several mechanisms regulating gene expression contribute to restore and reestablish cellular homeostasis so that plants can adapt and survive in adverse situations. MicroRNAs (miRNAs) play roles important in the transcriptional and post-transcriptional regulation of gene expression, emerging as a regulatory molecule key in the responses to plant stress, such as cold, heat, drought, and salt. This work is a comprehensive and large-scale miRNA analysis performed to characterize the miRNA population present in oil palm (*Elaeis guineensis Jacq.*) exposed to a high level of salt stress, to identify miRNA-putative target genes in the oil palm genome, and to perform an in silico comparison of the expression profile of the miRNAs and their putative target genes.

**Results:**

A group of 79 miRNAs was found in oil palm, been 52 known miRNAs and 27 new ones. The known miRNAs found belonged to 28 families. Those miRNAs led to 229 distinct miRNA-putative target genes identified in the genome of oil palm. miRNAs and putative target genes differentially expressed under salinity stress were then selected for functional annotation analysis. The regulation of transcription, DNA-templated, and the oxidation-reduction process were the biological processes with the highest number of hits to the putative target genes, while protein binding and DNA binding were the molecular functions with the highest number of hits. Finally, the nucleus was the cellular component with the highest number of hits. The functional annotation of the putative target genes differentially expressed under salinity stress showed several ones coding for transcription factors which have already proven able to result in tolerance to salinity stress by overexpression or knockout in other plant species.

**Conclusions:**

Our findings provide new insights into the early response of young oil palm plants to salinity stress and confirm an expected preponderant role of transcription factors - such as NF-YA3, HOX32, and GRF1 - in this response. Besides, it points out potential salt-responsive miRNAs and miRNA-putative target genes that one can utilize to develop oil palm plants tolerant to salinity stress.

**Supplementary Information:**

The online version contains supplementary material available at 10.1186/s12870-021-03296-9.

## Background

MicroRNAs (miRNAs) are small endogenous non-coding RNAs, usually 21 nucleotides long, known to impact almost all biological processes [1]. miRNAs play roles important in the transcriptional and post-transcriptional regulation of gene expression, emerging as a regulatory molecule key in the responses to plant stress, and the main components of miRNA show high conservation between species [2, 3].

The synthesis of plant miRNAs happens in the nucleus, where the encoded plant miRNA genes are processed by polymerase II to form miRNAs [4]. Long sequences of miRNAs are folded into a hairpin structure, known as primary miRNAs or pri-miRNAs, and are then cleaved by DCL1 to form short and incomplete double-stranded structures called pre-miRNAs [5]. Pre-miRNAs are cleaved by DCL1 or DCL4 to form a double-stranded miRNA known as miRNA dimer. Subsequently, the methyltransferase HEN1 carries out methylation of the 3′ end, and the miRNA is then transported to the cytoplasm by the plant homologous protein exportin-5 (HASTY, HST). Then the miRNA single-chain and the AGO protein form the RISC complex, binding to the complementary target mRNA to cleave or inhibit translation, obtaining negative regulation of the target gene [6, 7].

Several abiotic stressors, such as cold, heat, drought, and salt, affect the plant life cycle interfering with growth and productivity [8]. Several mechanisms regulating gene expression contribute to restore and reestablish cellular homeostasis so that plants can adapt and survive in adverse situations. miRNAs play a role important in regulating gene expression in response to stress conditions [1, 9].

Abiotic stresses upregulate some genes and downregulate others, depending on the role played by the gene. miRNAs responsive to water stress are present in *Oryza sativa* [10, 11], *Arabidopsis thaliana* [12], and *Medicago truncatula* [13, 14]. Sunkar and Zhu [15] showed that miR319c is positively regulated in Arabidopsis when subjected to cold stress but did not change when subjected to dehydration, salt, or ABA. Several miRNAs, such as miR156, miR159, miR167, miR171, miR319, and miR396, showed differential expression levels during the response to salt stress in *Arabidopsis* sp. [12] and *Zea mays* [16]. Using state-of-the-art sequencing technology (NGS) to identify miRNAs responsive to salt, Dong et al. [17] identified 104 differentially expressed miRNAs in soybean nodules under salt stress.

Some studies have reported miRNAs in oil palm. Nasaruddin et al. [18] found five new potential miRNA encoding sequences in a collection of 7284 oil palm EST sequences by a combined homology and structural analysis approach, having roles in regulating the auxin response, floral development, and basal transcription. Low et al. [19] applied a homology approach to identify 14 miRNAs in contigs assembled from sequences generated from the hypomethylated or gene-rich regions of *Elaeis guineensis* and *E. oleifera* genomes. Silva et al. [20] identified 57 mature miRNA in *E. guineensis* and 52 in *E oleifera*, respectively, and miRNA-target prediction revealed that most of these miRNA-putative target genes are transcription factors involved in the plant development process, particularly the regulation of root development. Ho et al. [21] investigated microRNA expression in oil palm female inflorescences at two stages of floral development corresponding to the emergence of floral meristems and the formation of floral organs, identifying 15 oil palm-specific miRNA candidates. Zheng et al. [22] identified 52 miRNAs in a study aiming to gain insights into the regulatory mechanisms of lipid and fatty acid (FA) metabolism in oil palm.

Oil palm (*Elaeis guineensis* Jacq.) is a source of vegetable oil that has great importance in many economic sectors. In Brazil, over 95% of the oil palm plantations are in the Amazon region. Due to environmental restrictions imposed on the use of the Amazon rainforest, and the logistical difficulties to flow the production to the main industrial centers in the country, there is a crescent demand of growers for the cultivation of oil palm in other geographic regions in the country. One must use irrigation in oil palm plantations outside the Amazon region in Brazil, mainly due to long periods of drought observed in these alternative regions with potential for oil palm cultivation [23, 24]. Between 25 and 30% of the irrigated land area in the World is affected by salt and is essentially commercially unproductive [25]. Because of that, Embrapa started working on a multi-omics approach to characterize morphophysiological and molecular responses of oil palm (*E. guineensis*) to drought and salinity stresses [26].

The current study is a follow-up to the characterization of the morphophysiological responses of oil palm plants to drought and salinity stress [24, 26]. We carried out a comprehensive, large-scale miRNA analysis to characterize the miRNA population present in oil palm exposed to a high level of salt stress, to identify miRNA-putative target genes in the oil palm genome, and to perform an in silico comparison of the expression profile of the miRNAs and their putative target genes.

## Results

As shown previously in Vieira et al. [24], the electrical conductivity (EC) of the saturation extract increased, and the water potential (Ψw) decreased in a NaCl dose-dependent manner. At the 12th day after imposing the stress (DAT), the EC values ranged from ±2 dS m^− 1^ (control plants) to ±45 dS m^− 1^ (stressed plants in substrate treated with 2.0 g of NaCl per 100 g of the substrate), while the Ψw values varied from zero to − 1.42 MPa, respectively (data not shown). There were no differences in the average evapotranspiration between the groups on day zero; however, when subjected to stress, the plants started to show differences in the evapotranspiration rates, remaining until the end of the experiment (data not shown).

**Fig. 1:**
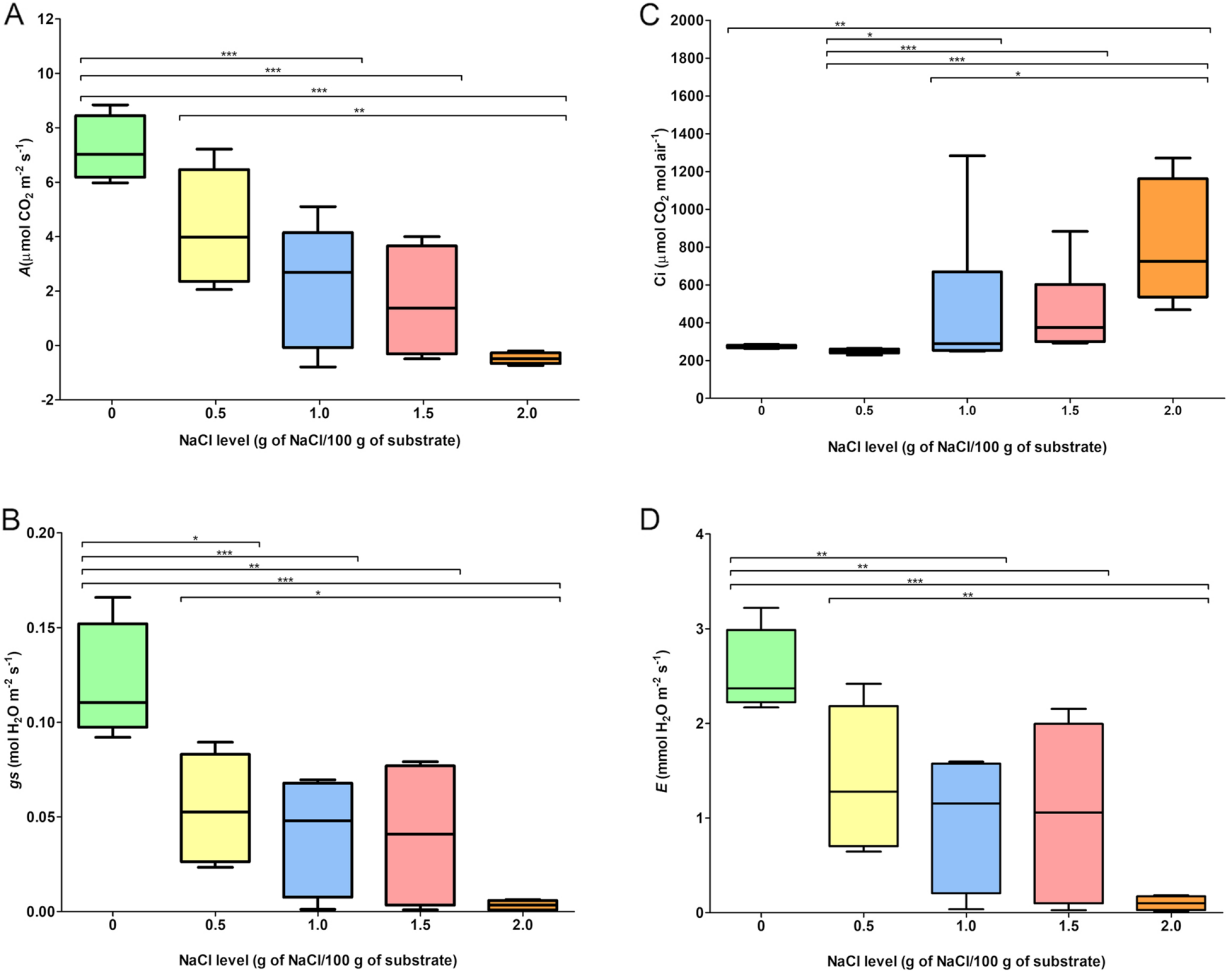
Box plots of the changes in gas exchange parameters in oil palm plants grown under increasing concentrations of NaCl in the cultivation substrate. **A** Net CO_2_ assimilation rate (*A*); (**B**) stomatal conductance rate to water vapor (*gs*); (**C**) intercellular CO_2_ concentration (*Ci*); and (**D**) transpiration rate (*E*). Medians and interquartile ranges are shown. The values represent the average of four replicates. The significance of differences was calculated using the Kruskal-Wallis test with *post-hoc* Dunn (*p* < 0.05). nsP > 0.05, non-significant comparisons were not shown in the graph; *P ≤ 0.05; **P ≤ 0.01; ***P ≤ 0.001

**Fig. 2:**
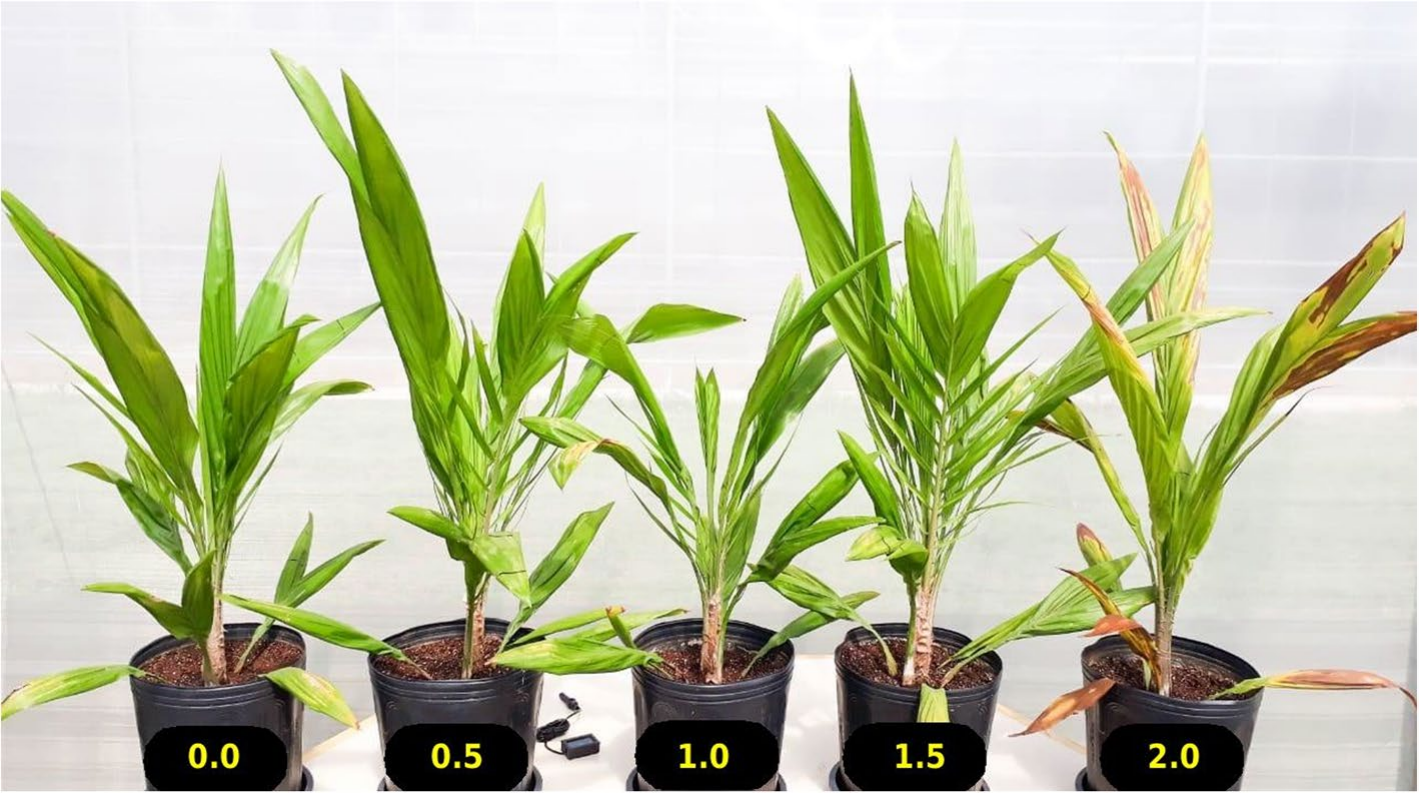
Young oil palm (*Elaeis guineensis*) plants at the bifid-sapling growth stage, after 12 days under salt stress. The value below each pot represents the amount of NaCl added to each 100 g of substrate, oven-dried at 105 °C for 24 h

At 12 DAT, it is visible a reduction in the rates of CO_2_ assimilation (*A*), stomatal conductance to water vapor (*gs*), and transpiration (*E*), which correlated with the amount of NaCl used (Fig. 1A, B, and D). On the other hand, the increase in intercellular CO_2_ concentration (*Ci*) also correlated with the amount of salt used (Fig. 1C). Stressed plants at the highest NaCl dose were already showing senescence of the leaves at 12 DAT (Fig. 2). Based on the morphophysiological responses of young oil palm plants to salinity stress (Figs. 1 and 2, and Vieira et al. [24]), both the control and the 2.0 g of NaCl per 100 g of the substrate treatments - which will be from now on referred as control and stressed treatments – were selected to the characterization of the microRNA and mRNA profiles.

### Identification of known and novel miRNAs, and differential expression analysis of miRNAs

The small RNA raw sequence data (9 fastq files) used in this study have been uploaded in the Sequence Read Archive (SRA) database of the National Center for Biotechnology Information under *Elaeis guineensis* microRNA_Drought and Salinity Stresses - BioProject number of PRJNA646488, BioSample SAMN12799239. All adapter-free small RNA sequences (from all nine fastq files) were concatenated into a single file and submitted for miRNA prediction using mireap version 0.2 and Shortstack version 3.4, generating 96 positive hits for potential miRNAs (data not shown). Concomitantly, all adapter-free small RNA sequences from control and salt-stressed samples (three replicates) were submitted to assemble and then mapped against the oil palm reference genome [27], generating 3384 positive hits (data not shown).

**Fig. 3:**
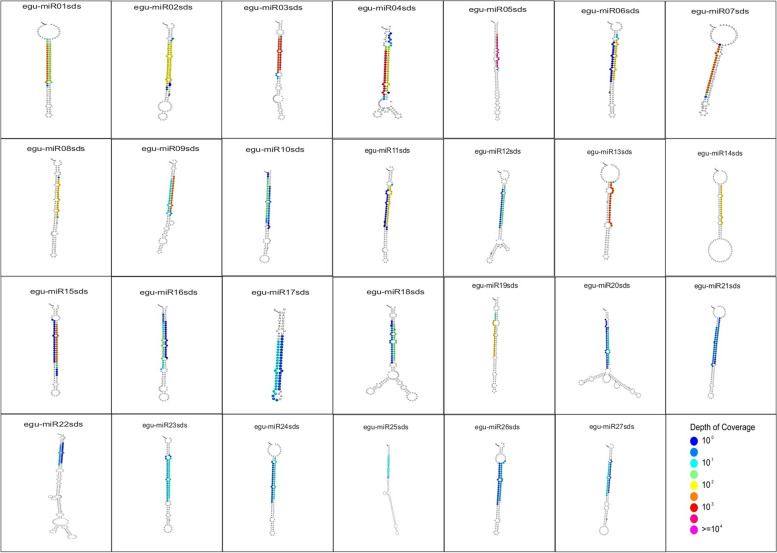
Structure of the 27 new miRNAs identified in oil palm (*Elaeis guineensis*) plants exposed to a high level of salt stress

A search in the database with the 3384 hits to the oil palm genome, using the database with the potential miRNAs, led to a total of 79 miRNAs, being 52 known miRNAs and 27 new ones (Fig. 3). The length of the 27 new ones ranges from 21 (24 miRNAs) to 22 (3 miRNAs) nucleotides (Supplementary Table 1). The genes of the 79 miRNAs identified in this study ranged from 68 to 285 bp in length and spread throughout all 16 chromosomes of the *E. guineensis* genome (Supplementary Table 2). Several miRNAs are present in more than one place in the genome, in different chromosomes, or at different positions in the same chromosome. Chromosomes 01, 04, and 08 had the highest miRNAs amount, 12, 11, and 11, respectively. Twenty-eight miRNAs got mapped to 28 unplaced scaffolds. The highest number of miRNAs in one unplaced scaffold was three, in scaffold NW_011551039.1 (Supplementary Table 2).

The new putative miRNA genes are between 68 and 267 bp in length (Supplementary Table 2). Regarding the location of these genes in the genome of *E. guineensis*, 20 of them were in intragenic and nine in intergenic regions (Table 1). Of the genes present inside genes, only the egu-miR16sds, egu-miR17sds, egu-miR22sds, and egu-miR26sds genes are in non-characterized ones (Table 1).

**Table 1:** New miRNAs identified in the oil palm (*Elaeis guineensis*) genome

miRNA Name	Shortstack cord	Location	Gene ID^a^	Gene description
egu-miR01sds	NC_025994.1:56908176–56,908,276	intragenic	LOC105039763	26S proteasome non-ATPase regulatory subunit 8 homolog A
egu-miR01sds	NW_011550933.1:850430–850,534	intragenic	LOC105061524	ubiquitin carboxyl-terminal hydrolase 3
egu-miR02sds	NC_025996.1:22888879–22,888,988	intragenic	LOC105042830	E3 ubiquitin-protein ligase HAKAI homolog
egu-miR03sds	NC_025997.1:33290362–33,290,493	intragenic	LOC105045464	hydroxymethylglutaryl-CoA lyase, mitochondrial
egu-miR04sds	NC_025998.1:32639960–32,640,067	intergenic	NA	NA
egu-miR05sds	NC_026000.1:8768033–8,768,169	intergenic	NA	NA
egu-miR06sds	NC_026001.1:23573097–23,573,199	intragenic	LOC105051421	plant UBX domain-containing protein 11
egu-miR07sds	NW_011552138.1:10926–10,993	intragenic	LOC105035654	probable peptide/nitrate transporter At3g43790
egu-miR07sds	NC_026007.1:3222601–3,222,690	intergenic	NA	NA
egu-miR08sds	NW_011551761.1:8894–9002	intragenic	LOC105035396	probable DNA helicase MCM8
egu-miR09sds	NW_011553407.1:22959–23,079	intragenic	LOC105036110	vesicle-associated membrane protein 721
egu-miR10sds	NW_011552437.1:17925–18,039	intergenic	NA	NA
egu-miR11sds	NC_025995.1:12779980–12,780,080	intragenic	LOC105040786	serine/threonine-protein phosphatase PP1
egu-miR12sds	NC_026007.1:23368340–23,368,461	intergenic	NA	NA
egu-miR13sds	NC_025996.1:46674015–46,674,106	intergenic	NA	NA
egu-miR14sds	NC_026002.1:30893315–30,893,427	intragenic	LOC105053431	E3 ubiquitin protein ligase DRIP2
egu-miR14sds	NC_026001.1:19506680–19,506,793	intergenic	NA	NA
egu-miR14sds	NC_025997.1:18353407–18,353,516	intergenic	NA	NA
egu-miR14sds	NW_011550939.1:17612–17,724	intragenic	LOC105031979	probable GDP-L-fucose synthase 1
egu-miR14sds	NW_011551049.1:134162–134,274	intragenic	LOC105033684	mevalonate kinase
egu-miR15sds	NC_026003.1:10876077–10,876,178	intragenic	LOC105053747	26S proteasome non-ATPase regulatory subunit 2 homolog A
egu-miR16sds	NC_025993.1:8597526–8,597,635	intragenic	LOC105039415	uncharacterized LOC105039415 – lncRNA
egu-miR17sds	NW_011551539.1:60051–60,120	intragenic	LOC105035181	uncharacterized LOC105035181 – lncRNA
egu-miR18sds	NC_025997.1:42070048–42,070,195	intergenic	NA	NA
egu-miR19sds	NC_026006.1:8032758–8,032,862	intragenic	LOC105057555	UPF0496 protein At5g66675-like
egu-miR20sds	NW_011551034.1:756120–756,299	intragenic	LOC105033525	histone-lysine N-methyltransferase ATXR2
egu-miR21sds	NC_025994.1:23652674–23,652,776	intragenic	LOC105038330	transcription factor VIP1
egu-miR22sds	NC_025996.1:56111754–56,112,020	intragenic	LOC105044280	uncharacterized LOC105044280 - protein coding
egu-miR23sds	NC_026002.1:13066673–13,066,777	intergenic	NA	NA
egu-miR24sds	NC_025996.1:46873993–46,874,094	intragenic	LOC105043881	ATP-dependent DNA helicase SRS2-like protein At4g25120
egu-miR25sds	NW_011551090.1:713220–713,417	intragenic	LOC105034003	ubiquitin receptor RAD23d
egu-miR26sds	NC_025993.1:37595103–37,595,199	intragenic	LOC105054928	uncharacterized LOC105054928 – protein coding
egu-miR27sds	NC_025996.1:40372686–40,372,807	intragenic	LOC105043419	shaggy-related protein kinase epsilon

^a^ BioProject PRJNA192219 and BioSample SAMN02981535, available at NCBI

The 52 known miRNAs found in this study belonged to 28 families. Among these families, the largest were miR156 and miR169 (5 members each), followed by miR166 and miR396 (4 members), miR159 and miR171 (3 members), miR168, miR319, miR393, miR395, miR399 and miR529 (2 members), and miR160, miR162, miR167, miR172, miR390, miR391, miR397, miR4 82, miR528, miR530, miR535, miR536, miR827, miR828, and miR2637 (one member).

Among the 79 known and novel miRNAs, 72 showed a significant (probability ≥0.95) different level of expression under saline stress; however, all were downregulated (Supplementary Table 2). These differentially expressed (DE) miRNAs had their expression level reduced in the range from 39.75 to 99.82%. In general, those DE miRNAs with their genes located in different regions in the genome did not present very distinct Log_2_(FC) values; the only exception was ppe-miR397 (Supplementary Table 2).

### Prediction and differential expression analysis of miRNA-putative target genes

The psRNA-Target online program, version 2, led to 425 positive hits as miRNA-putative target genes. When analyzing the mode of inhibition of these positive hits, the vast majority, 398, presented an mRNA cleavage mode and the remaining 27 a translation inhibition (Supplementary Table 3). It usually occurs because of some incompatibility around the center of the complementary region, as the central area is essential for cleavage [27].

Out of the 425 positive hits, there were 229 distinct putative target genes; based on the LOC Ids from the oil palm reference genome. Among these putative target genes, 150 were target to just one miRNA, and 79 were target to more than one miRNA - ranging from two to 36 miRNA per target gene (Supplementary Table 3).

The RNA-seq fastq files used in this study - from control and stressed plant samples, three replicates/treatment - are part of a group of 18 fastq files that have been uploaded in the Sequence Read Archive (SRA) database of the National Center for Biotechnology Information under *Elaeis guineensis* Transcriptome_Drought and Salinity Stresses - BioProject PRJNA573093, BioSample SAMN12799239. The transcriptome analysis showed that over 90% of the raw read pairs survived the preprocessing stage requiring a minimum average quality of reads ≥30 and the minimum length of reads ≥75 nucleotides. Over 95% of the high-quality read pairs mapped to the reference genome available at NCBI [28]. The reference genome has 29,567 genomic features of type ‘gene’ retrieved from 2781 ref. sequences in GCF_000442705.1_EG5_genomic.fna file; however, 4213 of these features had no aligned reads detected in any of the samples (Table 2).

**Table 2:** Statistics of RNA-Seq data from six samples of oil palm plants submitted to two treatments (0 and 2 g of NaCl per 100 g of the substrate); three replicates per treatment. Mapping to reference genome EG5 (BioProject PRJNA192219 and BioSample SAMN02981535) available at NCBI

Sample	Control_R1	Control_R2	Control_R3	Stressed_R1	Stressed_R2	Stressed_R3
**Input Read Pairs**	30,974,342	32,078,783	21,419,898	21,159,877	22,423,080	23,655,116
**Both Surviving Reads**	28,199,684 / 91.04%	28,906,033 / 90.11%	19,458,233 / 90.84%	19,244,188 / 90.95%	20,264,942 / 90.38%	21,601,656 / 91.32%
**Uniquely Mapped Reads**	26,504,692 / 93.989%	27,293,855 / 94.423%	17,156,499 / 88.171%	17,076,951 / 88.738%	17,363,794 / 85.684%	19,753,290 / 91.443%
**Average Mapped Length**	294.02	294.58	295.41	295.44	295.79	295.68
**Reads Mapped to Multiple Loci**	730,067 / 2.589%	654,325 / 2.264%	1,775,953 / 9.127%	1,621,318 / 8.425%	2,403,469 / 11.86%	1,270,546 / 5.882%
**Reads Aligned to Feature of Type ‘gene’**^a^	24,370,328 / 86.72%	25,185,822 / 87.68%	15,143,708 / 72.45%	14,791,141 / 72.16%	13,979,922 / 62.26%	17,597,354 / 78.43%
**Reads Not Aligned to Feature**	1,701,727 / 6.06%	1,665,256 / 5.80%	1,758,058 / 8.41%	2,081,861 / 10.16%	3,185,020 / 14.18%	1,930,248 / 8.60%
**Reads Aligned to More Than One Feature**	1,599,065 / 5.69%	1,432,045 / 4.99%	3,746,405 / 17.92%	3,421,162 / 16.69%	5,091,435 / 22.67%	2,683,016 / 11.96%

^a^ 29,567 genomic features of type ‘gene’, retrieved from 2781 ref. sequences in GCF_000442705.1_EG5_genomic.fna & 4213 features (14.25%) for with no aligned reads was detected in any of the samples

When comparing control against stressed plants, the pairwise differential expression analysis revealed that out of the 29,567 features from the *E. guineensis* genome [28], 5366 were DE at False Discovery Rate (FDR) < 0.05 (data not shown); being 2380 upregulated (Log_2_(FC) > 0) and 2986 downregulated (Log_2_(FC) < 0). By applying the same criteria for the differential expression analysis of the 229 distinct miRNA-putative target genes previously prospected (Supplementary Table 3), a group of 24 upregulated and 27 downregulated genes were identified (data not shown). These 51 DE putative target genes were integratively and functionally annotated (Table 3, Supplementary Table 4).

**Table 3:** Profile of differentially expressed miRNA and their differentially expressed target genes from oil palm. False discovery rate (FDR), counts per milllion (CPM), and fold change (FC)

Target Gene					miRNA			
ID^a^	Expression profile	FDR	log_**2**_FC	log_**2**_CPM	Name	Expression profile	Probability	log_**2**_FC
LOC105032827	UP	0.017	0.639	7.657	osa-miR159a.2	DOWN	0.96	−1.73
LOC105032890	UP	0.005	0.527	6.145	egu-miR12sds	DOWN	1.00	−5.49
LOC105034273	UP	0.007	0.710	5.881	ssp-miR827	DOWN	1.00	−2.93
LOC105035262	UP	0.020	0.500	6.502	atr-miR393	DOWN	0.98	−0.92
LOC105039459	UP	0.029	1.704	−0.968	bdi-miR529-5p	DOWN	1.00	−2.30
LOC105040914	UP	0.047	0.451	4.664	egu-miR03sds	DOWN	1.00	−2.23
LOC105043377	UP	0.000	1.670	5.767	ssp-miR827	DOWN	1.00	−2.93
LOC105043768	UP	0.008	0.530	7.110	atr-miR319e	DOWN	0.99	−1.93
LOC105043777	UP	0.001	0.591	6.996	vvi-miR828a	DOWN	1.00	−1.87
LOC105046708	UP	0.047	0.823	1.782	ata-miR166d-3p	DOWN	1.00	−2.51
LOC105046708	UP	0.047	0.823	1.782	atr-miR166b	DOWN	1.00	−2.76
LOC105046708	UP	0.047	0.823	1.782	osa-miR166i-3p	DOWN	1.00	−2.92
LOC105046708	UP	0.047	0.823	1.782	sly-miR166c-3p	DOWN	1.00	−1.76
LOC105047586	UP	0.000	1.863	0.439	bra-miR168a-5p	DOWN	1.00	−2.32
LOC105047586	UP	0.000	1.863	0.439	bra-miR168c-5p	DOWN	1.00	−1.83
LOC105048659	UP	0.013	0.705	3.253	egu-miR24sds	DOWN	0.95	−1.51
LOC105050858	UP	0.001	0.699	5.484	egu-miR11sds	DOWN	1.00	−4.26
LOC105051200	UP	0.001	1.303	2.815	atr-miR535	DOWN	1.00	−1.09
LOC105052568	UP	0.002	0.836	5.573	mtr-miR2673b	DOWN	1.00	−6.43
LOC105054175	UP	0.047	1.208	0.522	ata-miR396b-5p	DOWN	1.00	−2.53
LOC105054413	UP	0.000	1.031	5.014	osa-miR2118p	DOWN	1.00	−0.81
LOC105055689	UP	0.000	0.921	4.005	egu-miR18sds	DOWN	0.99	−2.72
LOC105056468	UP	0.000	1.366	4.716	ata-miR169d-5p	DOWN	0.98	−2.57
LOC105056609	UP	0.008	1.037	2.980	ata-miR167d-5p	DOWN	1.00	−2.55
LOC105059001	UP	0.016	0.514	4.698	egu-miR03sds	DOWN	1.00	−2.23
LOC105059776	UP	0.009	0.654	4.620	egu-miR27sds	NDE^b^	NDE	NDE
LOC105059810	UP	0.045	0.419	5.282	egu-miR09sds	DOWN	1.00	−3.22
LOC109505530	UP	0.000	2.950	−0.450	aof-miR536	DOWN	1.00	−3.55
LOC105031985	DOWN	0.000	−1.367	3.216	egu-miR18sds	DOWN	0.99	−2.72
LOC105032107	DOWN	0.006	−0.512	5.732	mes-miR393d	DOWN	1.00	−1.48
LOC105033129	DOWN	0.002	−0.622	4.658	egu-miR17sds	DOWN	0.99	−4.15
LOC105034164	DOWN	0.000	−1.053	5.714	ata-miR172b-3p	DOWN	0.98	−1.27
LOC105035561	DOWN	0.000	−1.006	3.118	ata-miR399a-3p	DOWN	1.00	−8.83
LOC105038401	DOWN	0.000	−0.902	3.311	egu-miR10sds	DOWN	1.00	−2.95
LOC105039220	DOWN	0.004	−0.617	4.451	aof-miR391	DOWN	1.00	−6.71
LOC105041147	DOWN	0.000	−0.834	3.888	egu-miR09sds	DOWN	1.00	−3.22
LOC105043694	DOWN	0.000	−1.320	4.891	aof-miR536	DOWN	1.00	−3.55
LOC105046087	DOWN	0.035	−0.596	7.237	egu-miR07sds	DOWN	1.00	−2.05
LOC105046096	DOWN	0.037	−0.493	4.096	egu-miR24sds	DOWN	0.95	−1.51
LOC105048141	DOWN	0.000	−1.356	2.377	egu-miR21sds	DOWN	0.98	−2.78
LOC105048606	DOWN	0.000	−2.036	2.656	ata-miR395c-3p	DOWN	1.00	−2.65
LOC105048718	DOWN	0.022	−0.434	5.325	egu-miR03sds	DOWN	1.00	−2.23
LOC105048722	DOWN	0.000	−0.948	3.893	egu-miR08sds	DOWN	1.00	−2.50
LOC105048783	DOWN	0.038	−1.133	2.064	ata-miR171a-3p	DOWN	0.99	−3.64
LOC105049211	DOWN	0.005	−0.757	3.512	ata-miR167d-5p	DOWN	1.00	−2.55
LOC105052116	DOWN	0.006	−0.515	5.757	egu-miR04sds	DOWN	1.00	−9.11
LOC105054869	DOWN	0.011	−0.624	3.628	egu-miR23sds	DOWN	0.96	−1.65
LOC105054987	DOWN	0.000	−0.990	5.245	mdm-miR171b	DOWN	1.00	−1.94
LOC105054987	DOWN	0.000	−0.990	5.245	vvi-miR171j	DOWN	0.99	−2.74
LOC105057798	DOWN	0.018	−1.098	2.516	osa-miR159a.2	DOWN	0.96	−1.73
LOC105058639	DOWN	0.001	−0.688	4.776	gma-miR482a-3p	NDE	NDE	NDE
LOC105059511	DOWN	0.000	−1.281	3.868	mdm-miR171b	DOWN	1.00	−1.94
LOC105059511	DOWN	0.000	−1.281	3.868	vvi-miR171j	DOWN	0.99	−2.74
LOC105060969	DOWN	0.000	−1.382	1.982	vvi-miR171j	DOWN	0.99	−2.74
LOC105061136	DOWN	0.007	−0.578	3.982	egu-miR18sds	DOWN	0.99	−2.72
LOC105061318	DOWN	0.002	−1.663	0.209	ata-miR396b-5p	DOWN	1.00	−2.53
LOC105061572	DOWN	0.038	−0.470	4.505	egu-miR07sds	DOWN	1.00	−2.05

^a^ BioProject PRJNA192219 and BioSample SAMN02981535, available at NCBI; ^b^ *NDE* non differentially expressed

### Integrating the expression profiles from DE miRNA-putative target genes and their respective DE miRNAs

By integrating the expression profiles of the upregulated miRNA-putative target genes and their respective miRNAs, twenty-one of them showed just one DE miRNA, one showed two (LOC105047586), and one showed four (LOC105046708) linked to the miRNA-target gene, while one had a non-differentially expressed miRNA linked to it (LOC105059776) (Table 3). On the other hand, by integrating the expression profiles of the downregulated miRNA-putative target genes and their respective miRNAs, twenty-five of them showed just one, and two showed two (LOC105054987, LOC105059511) DE miRNA linked to the miRNA-target gene, while one had a non-differentially expressed miRNA linked to it (LOC105058639) (Table 3).

Among the 40 DE miRNAs with DE miRNA-putative target genes, 28 had only one target gene, nine had three, and three had three (Table 3). DE miRNAs vvi-miR171j, gu-miR18sds, and egu-miR03sds had three distinct DE putative target genes presenting different profiles when submitted to salinity stress. Vvi-miR171j downregulated to 15% of its expression level in the control plants, while its putative target genes downregulated to 38 (LOC105060969), 41 (LOC105059511), and 50% (LOC105054987). DE miRNA egu-miR18sds also downregulated to 15% of its expression level in the control plants, while one of its putative target genes upregulated to 189% (LOC105055689) and two downregulated to 39 (LOC105031985) and 67% (LOC105061136). On the other hand, while egu-miR03sds downregulated to 21% of its expression level in the control plants, one of the putative target genes downregulated to 74% (LOC105048718) and the other two upregulated to 137 (LOC105040914) and 143% (LOC105059001) of their initial expression level (Table 3).

**Fig. 4:**
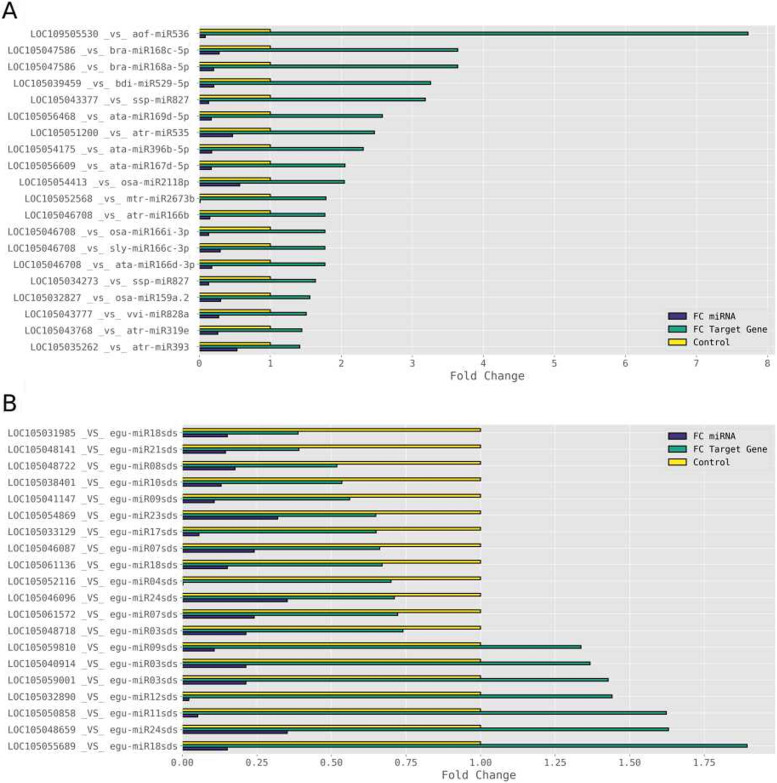
Expression profiles in fold change (FC) of the differentially expressed (DE) miRNAs and their respective DE target gene(s), resulted from submission of young oil palm plants to salinity stress, and in comparisson with the control treatment (FC = 1). A – DE known miRNAs and their upregulated putative target genes; and B - DE new miRNAs and their DE putative target genes

LOC109505530 was the miRNA-target gene that experienced the highest expression level increase in the leaf of oil palm plants due to saline stress. This gene is one of two found as targetted by aof-miR536, but the only one to upregulate due to this stress. The saline stress led aorf-miR536 to downregulate to less than 10% the level found in the control plants, while LOC109505530 upregulated to almost eight times its initial expression level (Fig. 4A). LOC105046708 is target of three distinct miRNAs (ata-miR166d-3p, sly-miR166c-3p, and osa-miR166i-3p) in the genome of oil palm. This gene experienced an increase of approximately 80% due to saline stress, while the miRNAs targetting it downregulated to between 13 and 18% the level found in the control plants (Fig. 4A).

All oil palm new miRNAs identified in this study downregulate due to saline stress. However, their differentially expressed putative target genes belonged to two groups according to their response to saline stress, seven upregulated and 13 downregulated (Fig. 4B). In the case of egu-miR13sds and egu-miR18sds, the fate of their respective putative target genes – each one had 3 - were completely distinct, with some upregulating and some downregulating due to saline stress (Fig. 4B).

### Functional annotation of the differencially expressed putative target genes

Among the 51 DE miRNA-putative target genes selected for functional annotation analysis, twenty had positive hits for biological process, 33 for molecular function, and eight for cellular component (Supplementary Table 4). The regulation of transcription, DNA-templated (GO:0006355), was the biological process with the highest number of hits, six, followed by the oxidation-reduction process (GO:0055114) with five. Protein binding (GO:0005515) and DNA binding (GO:0003677) were the molecular functions with the highest number of hits, six, five, respectively. The cellular component with the highest number of hits was the nucleus (GO:0005634), four, followed by the membrane (GO:0016021). Four genes had hits for domains from the GRAS family, a player important in gibberellin signaling [29].

The functional annotation analysis led to 18 known proteins, besides three lncRNA genes and three uncharacterized/unknown proteins. Among the 18 proteins there are six different kinds of transcription factors (LOC105054175, LOC105056468, LOC105046708, LOC105039459, LOC105048659, and LOC105043768). The remaining protein are: SPX-MFS proteins (LOC105043377, LOC105034273), JMJ16 protein (LOC105055689), TIR-1 like protein (LOC105035262), Delta4-sphingolipid-FADS-like protein (LOC105059001), PPR repeat-containing protein (LOC105032890), TPR repeat-containing protein (LOC105059776), Exportin 1-like protein (LOC105040914), 4-Oxalocrotonate Tautomerase protein (LOC105050858), Glycosyltransferase protein (LOC105056609), protein ORANGE (LOC105043777), and Actin protein (LOC105032827).

## Discussion

Among the few studies reporting miRNA in oil palm [18–22], none of them has studied the role of this type of macromolecule in the response of this species to salinity stress. So, to the best of our knowledge, this is the first report on the expression profile of oil palm miRNAs and their putative target genes when subjected to saline stress.

The 52 orthologous miRNAs identified in this study belong to 28 different families previously reported in oil palm as expressed in floral meristems - miR156, miR160, miR166, miR167, miR168, miR172, miR396, miR528, and miR535, by Ho et al. [21]; in the development of the mesocarp – miR156, miR395, and miR528, by Fang et al. [30]; and in shoot apical meristem, immature and mature flowers - miR156, miR159, and miR160, by Nasaruddin et al. [18] using ESTs from a study by Ho et al. [31].

The expression profile of the 79 miRNAs found in this study revealed that 72 of them downregulate in the salt-stressed plants, and the remaining had no significant differential expression (Supplementary Table 2). The genotypic specificity of the miRNAs behavior is usually evident since different genotypes express the same miRNA but at different levels [8, 32]. According to Sunkar et al. [8], a miRNA that presents negative regulation during stress probably targets positive regulators of stress tolerance, generating an accumulation of gene products.

Dong et al. [17] reported that miR159, miR169, and miR319 showed highly significant negative regulation in soybean nodules when subjected to salt stress. Other studies showed that miR393, miR394, miR396, and miR156 were responsive to this stress in *Arabidopsis thaliana*, *Zea mays*, *Populus tremula*, *Populus trichocarpa*, *Oryza sativa*, and *Glycine max* [15, 16, 33–35]. According to Ding et al. [16], miR159, miR160, miR162, miR164, miR166, miR167, miR168, miR171, miR319, miR395, miR396, and miR399 were responsive to the salt stress in the roots of corn when subjected to a 200 mM NaCl concentration. In *P. trichocarpa*, miR530 downregulate during salt stress, the same behavior observed in *Arabidopsis* spp. to miR396 [36]. These results corroborate with the miRNAs found in oil palm when subjected to salt stress.

Some studies show that a single miRNA can selectively regulate its targets in a non-linear dose-dependent manner, so preferred mRNA targets may vary over the developmental stages, depending on the level of expression of regulatory miRNAs [21, 37]. In Ho et al. [21], validation using RNA degradome data supports that a single miRNA may regulate multiple targets, and an mRNA may be regulated by more than one miRNA, suggesting a complex and fine-tuned interaction network between miRNAs and their targets at the post-transcriptional level. Ho and colleagues’ results corroborate with ours in oil palm subjected to salt stress, as miR171, egu-miR03sds and egu-miR18sds showed differential expression, and each one of them had three putative target genes also differentially expressed; the same is true to a putative target gene regulated by multiple miRNAs.

Many miRNAs play a role in the response of plants to abiotic stresses, such as salinity, through post-transcriptional regulation, which has been the focus of several studies [1, 8, 22, 38]. Different reports demonstrate that several miRNA-target genes are transcription factor mRNAs (TFs), indicating miRNA-dependent post-transitional regulation during the development and response to the environment [39, 40]. In plants, approximately 7–10% of the genes code for TFs at distinct moments, and dozen plant *TF* gene families precisely coordinate the spatial and temporal expression of downstream genes associated with abiotic stress [41, 42]. The present study showed that miRNAs miR166, miR169, miR319, miR396, miR529, and egu-miR24sds showed altered expression profiles in young oil palm plants subjected to salt stress; and, through functional analysis, that they regulate TFs transcript levels, which in turn affect the protein levels of the TFs.

The squamosa promoter-binding-like protein 17 homolog gene in oil palm (LOC105039459) is the putative target gene to miR529. While this miRNA underwent an 80% decrease in expression, its target gene showed a 70% increase in the apical leaf of young oil palm plants under very high salt stress. Squamosa promoter-binding (SBP) and SBP-Like (SPL) proteins are putative transcription factors having a plant-specific SBP domain consisting of 76 amino acids in length that regulates several biological processes, including salinity stress. Hou et al. [43] overexpressed the *VpSBP16* gene from grape (*Vitis vinifera*) in *A*. *thaliana* and observed an enhancement in the tolerance to salt and drought stress during seed germination, as well in seedlings and mature plants, by regulating SOS and ROS signaling cascades. On the other hand, the *OsSPL10* and *CaSBP12* genes negatively control salt tolerance in rice and pepper, respectively [29, 44].

The transcription factor bHLH143 homolog gene in oil palm (LOC105048659) is the putative target gene of egu-miR24sds. While this miRNA underwent a 65% decrease in expression, its putative target gene showed a 63% increase in the apical leaf of young oil palm plants under very high salt stress. The basic helix-loop-helix (bHLH) TFs are a large gene family in the plant genome, and some of these TFs regulate plant responses to abiotic stresses, including salt stress. The overexpression of *the SlbHLH22* gene in tomato plants increased the tolerance to drought and salinity stress by improving the ROS scavenging system, increasing osmotic potential, and enhanced the accumulation of secondary metabolites [45]. Qiu et al. [46] overexpressed the *MfbHLH38* gene - from the resurrection plant *Myrothamnus flabellifolia* - in Arabidopsis and observed enhanced tolerance to both drought and salinity stresses through increasing water retention ability, regulating osmotic balance, decreasing stress-induced oxidation damage, and possibly participated in ABA-dependent stress-responding pathway.

The GATA transcription factor 27 homolog gene in oil palm (LOC105043768) is the putative target gene to miR319. While this miRNA underwent a 74% decrease in expression, its target gene showed a 44% increase under very high salt stress. GATA TFs belong to one of the most conserved families of zinc-finger TFs [47]. Transcript abundance analysis using salt-sensitive and salt-tolerant rice genotypes indicated differential expression of GATA TF genes in response to various abiotic stresses such as salinity, drought, and exogenous ABA, suggesting inherent roles of diverse GATA factors in abiotic stress signaling [48]. Nutan et al. [49] have shown that the overexpression of the *OsGATA8* gene results in salinity tolerance in rice seedlings, as it maintains ion homeostasis and restricts membrane damage.

The homeobox-leucine zipper protein HOX32 homolog gene in oil palm (LOC105046708) is the putative target gene to four distinct miRNAs from the miR166 family. While these miRNAs underwent a 70–87% decrease in expression, its target gene showed a 78% increase in the apical leaf of young oil palm plants under very high salt stress. *Arabidopsis thaliana* has four distinct classes of homeodomain leucine zipper (HD-ZIP) transcription factors – HD-ZIPI to HD-ZIPIV – organized in multi-genes families [50]. Bhattacharjee et al. [51] carried out a functional analysis of two candidates stress-responsive HD-ZIP I class homeobox genes from rice, *OsHOX22*, and *OsHOX24*, and showed that these genes were highly upregulated under various abiotic stress conditions, including salinity stress, at different stages of development, including seedling, mature and reproductive stages. Besides that, Bhattacharjee and colleagues also overexpressed the *OsHOX24* gene in Arabidopsis plants showing that its overexpression does not result in a detectable difference in the phenotype and various growth parameters compared to the wild type under normal growth conditions; however, it does result in higher sensitivity to salinity stress.

The growth-regulating factor 10 homolog gene in oil palm (LOC105054175) is the putative target gene to miR396. While this miRNA underwent an 83% decrease in expression, its target gene showed a 131% increase in the apical leaf of young oil palm plants under very high salt stress. microRNA miR396 controls the expression of several growth-regulating factors (GRFs), and the GRF-miRNA396 regulatory module appears to be central to several developmental processes, including flower and seed formation, root development, and the coordination of growth processes under adverse environmental conditions, including salt stress [52–54]. Genetically modified creeping bentgrass (*Agrostis stolonifera*) overexpressing *Osa-miR396c*, a rice miRNA396 gene, showed enhanced salt tolerance associated with improved water retention, increased chlorophyll content, cell membrane integrity, and Na^+^ exclusion during high salinity exposure; however, they exhibited altered development [53]. RNA-sequencing analysis revealed that GRF1 and GRF3 regulate the expression of many clock core genes and genes with stress- and defense-related functions [55]. *AtGRF7* - a repressor of stress-responsive genes under non-stress conditions – suppresses *DREB2A* expression to preserve plant growth rate [56]. DREB2A is a TF whose transcriptional and post-translational activation increases osmotic stress tolerance in *Arabidopsis* [55]. *atgrf7* lost function mutants are more tolerant to drought and salinity stresses [52].

Nuclear factor Y (NF-Y) proteins are widespread in plants, animals, and other eukaryotes and are also known as *CCAAT* Binding Factor (CBF) or Heme Activator Protein (HAP); and they modulate the expression of downstream putative target genes via two main mechanisms [57]. The heterotrimer – NF-YA-YB-YC – binds to the CCAAT box present in the promoter region of the downstream putative target genes through NF-YA and regulates the expression of the putative target genes. The idea of NF-YA competing with TFs, and suppressing the formation of the NF-YB-YC-TF complex, was postulated [58]; however, according to Zhao et al. [57], there is still no direct molecular evidence to support it.

Different members of the NFY gene family, including NF-YA, are targets of the miR169 family, and studies have shown that overexpression of NF-YA in Arabidopsis increased the plant’s tolerance to salt stress, increasing the expression of abscisic acid [59]. A hypothetical model presented by Leyva-González et al. [58] proposes that in plants growing under non-stress conditions, NF-YA expression is low due to high levels of miR169 but sufficient to activate the transcription of genes which promoters contain the CCAAT box. In plants exposed to abiotic stress, NF-YA levels increase due to their transcriptional activation and to the reduction in the miR169 levels. Increased NF-YA levels repress early abiotic stress response genes probably by sequestering NF-YB-YC, creating a regulatory loop to arrest early responses that represent high energy and carbon costs, and participating in the activation of a late one.

Our results showed that the NF-YA3 homolog gene (LOC105056468) expression level in the apical leaf of salt-stressed young oil palm increased 158%, while miR169 had its expression level decreased to 17%. The gene that expresses the miR169 targetting the NF-YA3 homolog gene in oil palm is at two places in the oil palm genome, chromosomes 08 and 13, but only the one in the former chromosome differentially expressed under salinity stress (Supplementary Table 2). As the salinity stress reduces the amount of miR169 in the leaves of young oil palm plants, we postulate that more NF-YA3 would be available to compete with any NF-YB-YC-TF complex, resulting in more of the NF-YA-YB-YC complex, which could restore some of the main biological functions of this complex, such as drought tolerance.

A high concentration of soluble salts in the soil can directly affect plant growth in two distinct phases – osmotic and ionic -, whose duration and intensity vary according to the plant species and salt levels [60]. There is a rapid reduction in the osmotic potential in the osmotic stress phase that restricts water absorption and, therefore, reduces transpiration rates [60, 61]. Salinity in its first phase of salt stress is much similar to that of drought stress, and many common responses between salinity and drought stresses are also expected [62]. In the present study, the stressed plants showed a rate of evapotranspiration about half of the one in the control ones, which shows that the young oil palm plants were experiencing the osmotic stress at 12 DAT [24]. The ionic phase, on the contrary, occurs more slowly and depends not only on the saline concentration but also on the exposure time and on the plant’s capacity to accumulate or expel toxic ions [60, 63].

The young oil palm plants used in this study had been for 12 days under salinity stress when collecting leaves for the transcriptome characterization [24], which can be considered a short period when dealing with a perennial crop. Those plants had already shown - in the highest level of NaCl used - premature senescence, chlorosis, and necrosis of adult leaves, and consequently a reduction in the photosynthetic area available to support continued growth (Fig. 2). Such symptoms result from a high Na^+^ level in the plant that disrupts protein synthesis and interferes with enzyme activity [25]. In the case of these young oil palm plants, one can see an increase of almost 4X in Na^+^ and 2X in Cl^−^ in the absorption roots in the highest level of NaCl used, but not in the apical and basal leaves; showing that these plants were already starting to experience ionic stress [24].

## Conclusion

This comprehensive and large-scale miRNA analysis characterized the miRNA population present in the leaves of young oil palm plants exposed to a high level of salt stress, to identify miRNA-putative target genes in the oil palm genome, and to perform an in silico comparison of the expression profile of the miRNAs and their putative target genes, resulting in:

a) The identification of 79 miRNAs, 52 known miRNAs, and 27 new ones; 72 of them differentially expressed under salinity stress. The new ones received the names egu-miR(01to27)sds, where egu is the abbreviation of *Elaeis guineensis* and sds stands for salinity and drought stress;

b) The prediction of 229 distinct genes as the targets to these 79 miRNAs in the oil palm genome; 150 of them were target to just one miRNA and the remaining 79 to two or more. Fifty-one miRNA-putative target genes differentially expressed under salinity stress;

c) The functional annotation of 24 putative target genes upregulated under salinity stress. Among these genes, there were six that code for transcription factors and three for lncRNA; and.

d) The identification of potential targets genes – based upon evidence of a target gene-miRNA interaction under salinity stress - that can be tested as candidate genes to develop salinity stress tolerant oil palm plants. The development of salt-tolerant oil palm genotypes can come from overexpression or knock out of some of these miRNA or their respective putative target genes, either by a CRISPR/Cas genome editing strategy or by employing classic *Agrobacterium*- or biolistic-mediated genetic modification.

## Methods

### Plant material and growth conditions

The oil palm plants used in this study were clones regenerated in our lab out of embryogenic calluses obtained from leaves of an adult plant belonging to the *E. guineensis* genotype AM33, a Deli x Ghana from ASD Costa Rica (http://www.asd-cr.com). The protocols and procedures implemented to regenerate the plants are described in Corrêa et al. [64]. Plants were kept in black plastic pots (5 L), containing 1700 g of a mix of vermiculite, soil, and a commercial substrate (Bioplant®), in a 1:1:1 ratio on a dry basis, and fertilized using 2.5 g/L of the formula 20–20-20. Before starting the experiments, plants were standardized accordingly to the developmental stage, size, and number of leaves. The experiment was performed in a greenhouse at Embrapa Agroenergy (www.embrapa.br/en/agroenergia) in Brasília, DF, Brazil (S-15.732°, W-47.900°). The main environmental variables (temperature, humidity, and radiation) measured at a nearby meteorological station (S-15.789°, W-47.925°) fluctuated according to the weather conditions. The oil palm plants used in this study were in the growth stage known as “bifid saplings” when subjected to salt stress.

### Experimental design and saline stress

The experiment was carried out in March 2018 and consisted of five treatments (0.0, 0.5, 1.0, 1.5, and 2.0 g of NaCl per 100 g of substrate), with four replicates in a completely randomized design. For details regarding moisture content, field capacity, and electric conductivity in the substrate, determined preliminarily, see Vieira et al. [24].

To salinize the substrate, the amount of NaCl corresponding to the level to be applied to each treatment was dissolved in an amount of tap water standardized and calculated by the difference between the amount of water previously present in the fresh substrate and the amount of water retained for the substrate to reach field capacity. Applying the right amount of water to get the substrate field capacity was a means of ensuring that there was no extravasation of the solution and loss of Na^+^ or Cl^−^. Thus, the amount of salt added would remain in the substrate.

Plants were under stress for 12 days, with daily water maintenance by replacing the lost volume with tap water. The difference between total weight (TW) (container, soil, water added to reach field capacity, and plant weights, altogether) and the daily weight (DW) is equal to the amount of water necessary to replace daily water losses due to evapotranspiration. Such a procedure was essential to allow the same level of electric conductivity and water potential accordingly to the dose of salt added to the substrate.

### Gas exchange measurements

Gas exchange was measured on the middle third of the apical leaf, in a previously marked area, between 9:00 and 11:00 a.m. [24]. The parameters of leaf gas exchange [net CO_2_ assimilation rate (*A*), transpiration rate (*E*), stomatal conductance to water vapor (*gs*), and intercellular CO_2_ concentration (Ci)] were measured by a portable infrared gas analyzer LI-COR Mod. 6400XT (LI-COR, Lincoln, NE, USA) equipped with a measuring chamber (2 × 3 cm) with artificial light system LI-COR Mod. 6400-02B. The extracted data was provided by the OPEN software version 6.3. The block temperature was 25 °C, PAR was 1500 μmol/m^2^/s, the relative humidity of the air inside the measuring chamber was between 50 and 60%, the airflow index was 400 μmol/s, and the CO_2_ concentration was 400 ppm in the reference cell, using the model 6400–01 CO_2_ mixer with cylinder CO_2_ (7.5 g). After submitting the gas exchange data to the Kruskal-Wallis test, we applied the Dunn’s test (*p* < 0.05) to those data with significant differences between treatments.

### Transcriptomics

Apical leaves from three control and stressed plants (0.0 and 2.0 g of NaCl per 100 g of substrate), collected 12 days after imposition of the treatments (DAT), were immediately immersed in liquid nitrogen and then stored at − 80 °C until RNA extraction, library preparation, and sequencing.

#### Total RNA extraction and quality analysis, library preparation and sequencing

Total RNA was isolated from oil palm leaves using the Qiagen RNeasy® Plant Mini kit (QIAGEN, CA, USA) following the manufacturer’s protocol. RNA quantity and quality were measured using a Nanodrop Qubit 2.0 Fluorometer (Life Technologies, CA, USA) and an Agilent Bioanalyzer Model 2100 (Agilent Technologies, Palo Alto, CA). The GenOne Company (Rio de Janeiro, RJ, Brazil) performed the RNA-Seq using an Illumina HiSeq platform and the paired-end strategy. The Functional Genomics Center / ESALQ-USP (Piracicaba, SP, Brazil) performed the small RNAs sequencing using an Illumina HiSeq platform.

#### RNA-Seq data analysis

The OmicsBox version 1.3 [65] was employed to perform all RNA-Seq analyses. We used FastQC [66] and Trimmomatic [67] for quality control, filter reads, and remove low-quality bases. The oil palm reference genome [27] – files downloaded from NCBI (BioProject PRJNA192219; BioSample SAMN02981535) on October 2020 - was used to align the RNA-Seq data using default parameters from OmicsBox version 1.3 through software STAR [68]. The default parameters from OmicsBox version 1.3 through HTSeq version 0.9.0 were employed to quantify expression at the gene or transcript level [69]. The pairwise differential expression analysis between experimental conditions (Control vs. Stressed) was performed through edgeR version 3.28.0 [70], applying a simple design and an exact statistical test without a filter for low counts genes.

### miRNAs data analysis

The small RNA raw data was submitted to the cutadapt software version 2.7 [71], generating adapter-free small RNA reads 20–24 nucleotides long. The Rfam version 12.0 database was used to remove contaminants, followed by mapping to the oil palm reference genome [27] using Bowtie2 [72].

All adapter-free small RNA sequences (stressed and control) were concatenated into a single file for miRNA prediction. The prediction was then made using mireap version 0.2 (https://sourceforge.net/projects/mireap) and Shortstack version 3.4 (https://github.com/MikeAxtell/ShortStack), independently or in an association. Both programs generate clusters of sequences lined up in genomic regions. Ideally, these clusters indicate the genomic location and the miRNA precursor, mature miRNA, and miRNA* sequences. Shortstack also analyzes precursor and hairpin metrics formed according to parameters established by Axtell and Meyers and classifies them in Y (confirmed miRNA) or N1-N15, where N15 means that the candidate has all the correct metrics, but the miRNA* is absent [73]. The clusters formed by the mireap were analyzed by Shortack to obtain the classifications of each miRNA. StrucVis (https://github.com/MikeAxtell/strucVis) was used in sequences classified as Y or N15 by ShortStack and/or ShortStack-mireap for structural evaluation of miRNA. Finally, manual curation was made of all miRNAs classified as Y and N15. The length of the strings, the predicted structure of the hairpin, and the annotation by homology were evaluated (miRBase - http://www.mirbase.org/search.shtml).

The prediction of miRNA-putative target genes was performed using the psRNA-Target online program, version 2 (https://bio.tools/psrnatarget), with the following parameters: 5 of top targets, 5 expectation, 1 Penalty for other mismatches. For the analysis of differential expression of miRNAs, we used the NOISeq R package [74]. For this, the individual counts of each sample were used as input. The genes that showed *p* values ≥0.95 were designated as differentially expressed.

To functionally annotate the differentially expressed miRNA-putative target genes we used the LOC id to get to the protein sequence at NCBI, and then submitted it to the InterProScan search at InterPro (http://www.ebi.ac.uk/interpro/) [75].

## Supplementary Information

**Additional file 1: Supplementary Table 1.**Name, sequence and size of oil palm miRNAs predicted using mireap version 0.2 and Shortstack version 3.4, independently or in association, from the smallRNA raw data obtained from six samples of oil palm plants submitted to two treatments (0 and 2 g of NaCl per 100 g of the substrate); three replicates per treatment.

**Additional file 2: Supplementary Table 2.**Name of the miRNA, localization and size of oil palm miRNA gene, and differential expression profile of the putative miRNA genes in oil palm plants under salinity stress (2 g of NaCl per 100 g of the substrate), in comparison to control plants. FC – Fold change.

**Additional file 3: Supplementary Table 3.**Prediction of oil palm miRNAs-putative target genes using psRNA-Target online program, version 2, and the 79 miRNAs predicted in this study.

**Additional file 4: Supplementary Table 4.** Gene ontology (GO) classification of the oil palm gene targets for orthologous and new miRNAs prospected under salinity stress. The mRNA-putative target genes were classified into biological process, molecular function, and cellular component at the second level of GO classification.

## Data Availability

The smallRNA raw sequence data used in this study have been uploaded in the SRA database of the NCBI under *Elaeis guineensis* microRNA_Drought and Salinity Stresses - BioProject PRJNA646488 (SUB7775347), BioSample SAMN12799239 (SUB6325749), SRA submission SUB7897143 (accessions from SRR12424937 to SRR12424945), and will be available after publication of this study. All RNA-seq fastq files used in this study have been uploaded in the SRA database of the NCBI under *Elaeis guineensis* Transcriptome_Drought and Salinity Stresses - BioProject PRJNA573093 (SUB6324604), BioSample SAMN12799239 (SUB6325749), SRA submission SUB6335775 (accessions from SRR10219424 to SRR10219441), and will be available after publication of this study. The data-sets used and/or analyzed during the current study are available from the corresponding author on reasonable request.

## Abbreviations

Embrapa: Brazilian agricultural research corporation
PGBV: Graduate program in plant biotechnology
UFLA: Federal university of Lavras
TF: Transcription factors
DE: Differentially expressed
FDR: False discovery rate
DEG: Differentially expressed gene
ROS: Reactive oxygen species
CRISPR: Clustered regularly interspaced short palindromic repeats

## References

[1] Wang, J., Meng, X., Dobrovolskaya, O. B., Orlov, Y. L., & Chen, M. Non-coding RNAs and their roles in stress response in plants. Genomics Proteomics Bioinformatics 2017; 15(5), 301–312. https://doi.org/10.1016/j.gpb.2017.01.007. 10.1016/j.gpb.2017.01.007 PMC5673675 29017967

[2] Ayubov, M. S., Mirzakhmedov, M. H., Sripathi, V. R., Buriev, Z. T., Ubaydullaeva, K. A., Usmonov, D. E., Norboboyeva, R. B., Emani, C., Kumpatla, S. P., & Abdurakhmonov, I. Y. Role of MicroRNAs and small RNAs in regulation of developmental processes and agronomic traits in Gossypium species. Genomics. 2019; 111(5), 1018–1025. https://doi.org/10.1016/j.ygeno.2018.07.012. 10.1016/j.ygeno.2018.07.012 30026106

[3] Xu, X.W., Zhou, X.H., Wang, R.R. et al. Functional analysis of long intergenic non-coding RNAs in phosphate-starved rice using competing endogenous RNA network. 2016; Sci Rep 6, 20715. https://doi.org/10.1038/srep20715. 10.1038/srep20715 PMC4748279 26860696

[4] Budak, H., & Akpinar, B. A. Plant miRNAs: biogenesis, organization and origins. Funct Integr Genomics 2015; 15(5), 523–531. https://doi.org/10.1007/s10142-015-0451-2. 10.1007/s10142-015-0451-2 26113396

[5] Kim, Young Kook, Boseon Kim, and V. Narry Kim. “Re-Evaluation of the Roles of DROSHA, Exportin 5, and DICER in microRNA Biogenesis.” Proc Natl Acad Sci U S A 2016*;* 113(13): E1881–E1889. https://doi.org/10.1073/pnas.1602532113. 10.1073/pnas.1602532113 PMC4822641 26976605

[6] Chuammitri, P., Vannamahaxay, S., Sornpet, B., Pringproa, K., & Patchanee, P. Detection and characterization of microRNA expression profiling and its target genes in response to canine parvovirus in Crandell Reese Feline Kidney cells. 2020; PeerJ, *8*, e8522. https://doi.org/10.7717/peerj.8522. 10.7717/peerj.8522 PMC7023829 32095352

[7] Sun, X., Lin, L., & Sui, N. Regulation mechanism of microRNA in plant response to abiotic stress and breeding. Mol Biol Rep 2019; 46(1), 1447–1457. https://doi.org/10.1007/s11033-018-4511-2. 10.1007/s11033-018-4511-2 30465132

[8] Sunkar, R., Chinnusamy, V., Zhu, J., & Zhu, J. K. Small RNAs as big players in plant abiotic stress responses and nutrient deprivation. Trends Plant Sci 2007; 12(7), 301–309. https://doi.org/10.1016/j.tplants.2007.05.001. 10.1016/j.tplants.2007.05.001 17573231

[9] Xu, J., Hou, Q. M., Khare, T., Verma, S. K., & Kumar, V. Exploring miRNAs for developing climate-resilient crops: a perspective review. Sci Total Environ 2019; 653, 91–104. https://doi.org/10.1016/j.scitotenv.2018.10.340. 10.1016/j.scitotenv.2018.10.340 30408672

[10] Zhao, B., Liang, R., Ge, L., et al. Identification of drought-induced microRNAs in rice. Biochem Biophys Res Commun*.* 2007*;* 354(2), 585–590. DOI: https://doi.org/10.1016/j.bbrc.2007.01.022. 10.1016/j.bbrc.2007.01.022 17254555

[11] Zhou, Liguo et al. “Genome-wide identification and analysis of drought-responsive microRNAs in Oryza Sativa.” J Exp Bot 2010*;* 61(15): 4157–4168. https://doi.org/10.1093/jxb/erq237. 10.1093/jxb/erq237 20729483

[12] Liu, H. H., Tian, X., Li, Y. J., Wu, C. A., & Zheng, C. C. Microarray-based analysis of stress-regulated microRNAs in *Arabidopsis thaliana*. RNA*. 2008; 14*(5), 836–843. https://doi.org/10.1261/rna.895308. 10.1261/rna.895308 PMC2327369 18356539

[13] Wang, T., Chen, L., Zhao, M., Tian, Q., & Zhang, W. H. Identification of drought-responsive microRNAs in Medicago truncatula by genome-wide high-throughput sequencing. BMC Genomics 2011; 12, 367. https://doi.org/10.1186/1471-2164-12-367. 10.1186/1471-2164-12-367 PMC3160423 21762498

[14] Li, C., Li, Y., Bai, L., Chaoxing, H.E., Xianchang, Y.U. Dynamic expression of miRNAs and their targets in the response to drought stress of grafted cucumber seedlings. Hortic Plant J 2016; 2, 41–49. http://dx.doi.org/10.1016/j.hpj.2016.02.002

[15] Sunkar, R., and Zhu, J.K. Novel and stress regulated microRNAs and other small RNAs from Arabidopsis w inside box sign. Plant Cell 2004*;* 16(8), 2001–2019. https://doi.org/10.1105/tpc.104.022830. 10.1105/tpc.104.022830 PMC519194 15258262

[16] Ding, D., Zhang, L., Wang, H., Liu, Z., Zhang, Z., & Zheng, Y. Differential expression of miRNAs in response to salt stress in maize roots. Ann Bot 2009; 103(1), 29–38. https://doi.org/10.1093/aob/mcn205. 10.1093/aob/mcn205 PMC2707283 18952624

[17] Dong, Z., Shi, L., Wang, Y., Chen, L., Cai, Z., Wang, Y., Jin, J., & Li, X. Identification and dynamic regulation of microRNAs involved in salt stress responses in functional soybean nodules by high-throughput sequencing. Int J Mol Sci 2013; 14(2), 2717–2738. https://doi.org/10.3390/ijms14022717. 10.3390/ijms14022717 PMC3588011 23358256

[18] Md Nasaruddin N, Harikrishna K, Othman R, Hoon L, Ann Harikrishna J (2007). Computational prediction of microRNAs from oil palm (Elaeis guineensis Jacq.) expressed sequence tags. Asia Pac J Mol Biol Biotechnol.

[19] Low, E. T., Rosli, R., Jayanthi, N., Mohd-Amin, A. H., Azizi, N., Chan, K. L., Maqbool, N. J., Maclean, P., Brauning, R., McCulloch, A., Moraga, R., Ong-Abdullah, M., & Singh, R. Analyses of hypomethylated oil palm gene space. PLoS One 2014; 9(1), e86728. https://doi.org/10.1371/journal.pone.0086728. 10.1371/journal.pone.0086728 PMC3907425 24497974

[20] da Silva, A. C., Grativol, C., Thiebaut, F., Hemerly, A. S., & Ferreira, P. C. Computational identification and comparative analysis of miRNA precursors in three palm species. Planta. 2016; 243(5), 1265–1277. https://doi.org/10.1007/s00425-016-2486-6. 10.1007/s00425-016-2486-6 26919984

[21] Ho, H., Gudimella, R., Ong-Abdullah, M. et al. Expression of microRNAs during female inflorescence development in African oil palm (*Elaeis guineensis* Jacq.). Tree Genet Genomes*.* 2017*;* 13, 35. https://doi.org/10.1007/s11295-017-1120-5.

[22] Zheng, Y., Chen, C., Liang, Y., Sun, R., Gao, L., Liu, T., & Li, D. Genome-wide association analysis of the lipid and fatty acid metabolism regulatory network in the mesocarp of oil palm (Elaeis guineensis Jacq.) based on small noncoding RNA sequencing. Tree Physiol 2019; 39(3), 356–371. https://doi.org/10.1093/treephys/tpy091. 10.1093/treephys/tpy091 30137626

[23] Abrapalma. Diagnóstico da Produção Sustentável da Palma de Óleo. 2018; http://www.abrapalma.org/pt/wpcontent/uploads/2018/06/DIAGNOSTICO_PALMA.pdf

[24] Vieira, L. R., et al. “Morphophysiological responses of young oil palm plants to salinity stress”. Pesq Agrop Brasileira 2020; v.55, e01835. https://doi.org/10.1590/S1678-3921.pab2020.v55.01835.

[25] Carillo, P., Annunziata, M. G., Pontecorvo, G., Fuggi, A., Woodrow, P. Salinity Stress and Salt Tolerance, Abiotic Stress in Plants - Mechanisms and Adaptations, Prof. Arun Shanker (Ed.), ISBN: 978–953–307-394-1, InTech, 2011. Available from: http://www.intechopen.com/books/abiotic-stress-in-plants-mechanisms-and-adaptations/salinity-stress-and-salt-tolerance

[26] Vieira, L. R. Morphophysiological, Metabolomic and transcrytomic responses of oil palm (Elaeis guineensis) to drought and salinity stresses. 2019. 158 p. d.Sc. Thesis – Universidade federal de Lavras, Lavras, MG, Brazil. http://repositorio.ufla.br/jspui/handle/1/46074

[27] Brodersen, P., Sakvarelidze-Achard, L., Bruun-Rasmussen, M., Dunoyer, P., Yamamoto, Y. Y., Sieburth, L., & Voinnet, O. Widespread translational inhibition by plant miRNAs and siRNAs. Science 2008; *320*(5880), 1185–1190. https://doi.org/10.1126/science.1159151. 10.1126/science.1159151 18483398

[28] Singh, R., Ong-Abdullah, M., Low, E. T., Manaf, M. A., Rosli, R., Nookiah, R., Ooi, L. C., Ooi, S. E., Chan, K. L., Halim, M. A., Azizi, N., Nagappan, J., Bacher, B., Lakey, N., Smith, S. W., He, D., Hogan, M., Budiman, M. A., Lee, E. K., DeSalle, R., Sambanthamurthi, R. Oil palm genome sequence reveals divergence of interfertile species in old and new worlds. Nature. 2013; 500(7462), 335–339. https://doi.org/10.1038/nature12309. 10.1038/nature12309 PMC3929164 23883927

[29] Zhang, H. X., Zhu, W. C., Feng, X. H., Jin, J. H., Wei, A. M., & Gong, Z. H. Transcription factor *CaSBP12* negatively regulates salt stress tolerance in pepper (*Capsicum annuum* L.). Int J Mol Sci. 2020; *21*(2), 444. https://doi.org/10.3390/ijms21020444. 10.3390/ijms21020444 PMC7013666 31936712

[30] Fang L, Liang Y, Li D, Cao X, Zheng Y (2013). Dynamic expression analysis of miRNAs during the development process of oil palm mesocarp. Plant Sci J.

[31] Ho, C. L., Kwan, Y. Y., Choi, M. C., Tee, S. S., Ng, W. H., Lim, K. A., Lee, Y. P., Ooi, S. E., Lee, W. W., Tee, J. M., Tan, S. H., Kulaveerasingam, H., Alwee, S. S., & Abdullah, M. O. Analysis and functional annotation of expressed sequence tags (ESTs) from multiple tissues of oil palm (*Elaeis guineensis* Jacq.). BMC Genomics. 2007; 8, 381. https://doi.org/10.1186/1471-2164-8-381. 10.1186/1471-2164-8-381 PMC2222642 17953740

[32] Mica, E., Gianfranceschi, L., & Pè, M. E. Characterization of five microRNA families in maize. J Exp Bot 2006; 57(11), 2601–2612. https://doi.org/10.1093/jxb/erl013. 10.1093/jxb/erl013 16820394

[33] Gao, P., Bai, X., Yang, L., Lv, D., Pan, X., Li, Y., Cai, H., Ji, W., Chen, Q., & Zhu, Y. Osa-MIR393: a salinity- and alkaline stress-related microRNA gene. Mol Biol Rep 2011; 38(1), 237–242. https://doi.org/10.1007/s11033-010-0100-8. 10.1007/s11033-010-0100-8 20336383

[34] Guo Q, Li L, Zhao K, Yao W, Cheng Z, Zhou B, Jiang T. Genome-wide analysis of poplar SQUAMOSA-promoter-binding protein (SBP) family under salt stress. Forests*.* 2021; 12(4):413. https://doi.org/10.3390/f12040413.

[35] Lu, S., Sun, Y. H., & Chiang, V. L. Stress-responsive microRNAs in Populus. Plant J 2008; 55(1), 131–151. https://doi.org/10.1111/j.1365-313X.2008.03497.x. 10.1111/j.1365-313X.2008.03497.x 18363789

[36] Sunkar R. MicroRNAs with macro-effects on plant stress responses. Semin Cell Dev Biol 2010; 21(8), 805–811. https://doi.org/10.1016/j.semcdb.2010.04.001. 10.1016/j.semcdb.2010.04.001 20398781

[37] Lim, L. P., Lau, N. C., Garrett-Engele, P., Grimson, A., Schelter, J. M., Castle, J., Bartel, D. P., Linsley, P. S., & Johnson, J. M. Microarray analysis shows that some microRNAs downregulate large numbers of target mRNAs. Nature. 2005; 433(7027), 769–773. https://doi.org/10.1038/nature03315. 10.1038/nature03315 15685193

[38] Xu, J., Chen, Q., Liu, P., Jia, W., Chen, Z., & Xu, Z. Integration of mRNA and miRNA analysis reveals the molecular mechanism underlying salt and alkali stress tolerance in tobacco. Int J Mol Sci 2019; 20(10), 2391. https://doi.org/10.3390/ijms20102391. 10.3390/ijms20102391 PMC6566703 31091777

[39] Cheng, Y., & Long, M. A cytosolic NADP-malic enzyme gene from rice (Oryza sativa L.) confers salt tolerance in transgenic Arabidopsis. Biotechnol Lett 2007; 29(7), 1129–1134. https://doi.org/10.1007/s10529-007-9347-0. 10.1007/s10529-007-9347-0 17516134

[40] Prashanth, S. R., Sadhasivam, V., & Parida, A. Over expression of cytosolic copper/zinc superoxide dismutase from a mangrove plant Avicennia marina in indica rice var Pusa Basmati-1 confers abiotic stress tolerance. Transgenic Res 2008; 17(2), 281–291. https://doi.org/10.1007/s11248-007-9099-6. 10.1007/s11248-007-9099-6 17541718

[41] Javed, T., Shabbir, R., Ali, A., Afzal, I., Zaheer, U., & Gao, S. J. Transcription factors in plant stress responses: challenges and potential for sugarcane improvement. Plants 2020; *9*(4), 491. https://doi.org/10.3390/plants9040491. 10.3390/plants9040491 PMC7238037 32290272

[42] Wang, J., Ye, Y., Xu, M. et al. Roles of the SPL gene family and miR156 in the salt stress responses of tamarisk (*Tamarix chinensis*). BMC Plant Biol 2019*;* 19, 370. https://doi.org/10.1186/s12870-019-1977-6. 10.1186/s12870-019-1977-6 PMC6704519 31438851

[43] Hou, H., Jia, H., Yan, Q., & Wang, X. Overexpression of a SBP-box gene (VpSBP16) from Chinese wild Vitis species in Arabidopsis improves salinity and drought stress tolerance. Int J Mol Sci 2018; 19(4), 940. https://doi.org/10.3390/ijms19040940. 10.3390/ijms19040940 PMC5979544 29565279

[44] Lan, T., Zheng, Y., Su, Z., Yu, S., Song, H., Zheng, X., Lin, G., & Wu, W. *OsSPL10*, a SBP-box gene, plays a dual role in salt tolerance and trichome formation in rice (*Oryza sativa* L.). G3 2019; 9(12), 4107–4114. https://doi.org/10.1534/g3.119.400700. 10.1534/g3.119.400700 PMC6893181 31611344

[45] Waseem, M., Rong, X., & Li, Z. Dissecting the role of a basic Helix-loop-Helix transcription factor, *SlbHLH22*, under salt and drought stresses in transgenic *Solanum lycopersicum* L Front Plant Sci 2019; 10, 734. https://doi.org/10.3389/fpls.2019.00734. 10.3389/fpls.2019.00734 PMC6558761 31231412

[46] Qiu, J. R., Huang, Z., Xiang, X. Y., Xu, W. X., Wang, J. T., Chen, J., Song, L., Xiao, Y., Li, X., Ma, J., Cai, S. Z., Sun, L. X., & Jiang, C. Z. MfbHLH38, a Myrothamnus flabellifolia bHLH transcription factor, confers tolerance to drought and salinity stresses in Arabidopsis. BMC Plant Biol 2020; 20(1), 542. https://doi.org/10.1186/s12870-020-02732-6. 10.1186/s12870-020-02732-6 PMC7709435 33267774

[47] Reyes, J. C., Muro-Pastor, M. I., & Florencio, F. J. The GATA family of transcription factors in Arabidopsis and rice. Plant Physiol 2004; 134(4), 1718–1732. https://doi.org/10.1104/pp.103.037788. 10.1104/pp.103.037788 PMC419845 15084732

[48] Gupta, P., Nutan, K. K., Singla-Pareek, S. L., & Pareek, A. Abiotic stresses cause differential regulation of alternative splice forms of GATA transcription factor in Rice. Front Plant Sci 2017; 8, 1944. https://doi.org/10.3389/fpls.2017.01944. 10.3389/fpls.2017.01944 PMC5693882 29181013

[49] Nutan, K.K., Singla-Pareek, S.L., Pareek, A. The Saltol QTL-localized transcription factor OsGATA8 plays an important role in stress tolerance and seed development in Arabidopsis and rice, J Exp Bot. 2020; V. 71, Issue 2, 7 January 2020, Pages 684–698, https://doi.org/10.1093/jxb/erz368. 10.1093/jxb/erz368 31613368

[50] Brandt R, Cabedo M, Xie Y, Wenkel S (2014). Homeodomain leucine-zipper proteins and their role in synchronizing growth and development with the environment. J Integr Plant Biol.

[51] Bhattacharjee, A., Khurana, J. P., & Jain, M. Characterization of Rice Homeobox genes, OsHOX22 and OsHOX24, and over-expression of OsHOX24 in transgenic Arabidopsis suggest their role in abiotic stress response. Front Plant Sci 2016; 7, 627. https://doi.org/10.3389/fpls.2016.00627. 10.3389/fpls.2016.00627 PMC4862318 27242831

[52] Omidbakhshfard, M. A., Proost, S., Fujikura, U., & Mueller-Roeber, B. Growth-regulating factors (GRFs): a small transcription factor family with important functions in plant biology. Mol Plant 2015; 8(7), 998–1010. https://doi.org/10.1016/j.molp.2015.01.013. 10.1016/j.molp.2015.01.013 25620770

[53] Yuan, S., Zhao, J., Li, Z., Hu, Q., Yuan, N., Zhou, M., Xia, X., Noorai, R., Saski, C., Li, S., & Luo, H. MicroRNA396-mediated alteration in plant development and salinity stress response in creeping bentgrass. Horticulture Res 2019; 6, 48. https://doi.org/10.1038/s41438-019-0130-x. 10.1038/s41438-019-0130-x PMC6491569 31069081

[54] Rossmann, S., Richter, R., Sun, H., Schneeberger, K., Töpfer, R., Zyprian, E., & Theres, K. Mutations in the miR396 binding site of the growth-regulating factor gene VvGRF4 modulate inflorescence architecture in grapevine. Plant J 2020; 101(5), 1234–1248. https://doi.org/10.1111/tpj.14588. 10.1111/tpj.14588 31663642

[55] Piya, S., Liu, J., Burch-Smith, T., Baum, T. J., & Hewezi, T. A role for Arabidopsis growth-regulating factors 1 and 3 in growth-stress antagonism. J Exp Bot 2020; 71(4), 1402–1417. https://doi.org/10.1093/jxb/erz502. 10.1093/jxb/erz502 PMC7031083 31701146

[56] Kim, J. S., Mizoi, J., Kidokoro, S., Maruyama, K., Nakajima, J., Nakashima, K., Mitsuda, N., Takiguchi, Y., Ohme-Takagi, M., Kondou, Y., Yoshizumi, T., Matsui, M., Shinozaki, K., & Yamaguchi-Shinozaki, K. Arabidopsis growth-regulating factor7 functions as a transcriptional repressor of abscisic acid- and osmotic stress-responsive genes, including DREB2A. Plant Cell 2012; 24(8), 3393–3405. https://doi.org/10.1105/tpc.112.100933. 10.1105/tpc.112.100933 PMC3462639 22942381

[57] Zhao H, Wu D, Kong F, Lin K, Zhang H, Li G. The *Arabidopsis thaliana* nuclear factor Y transcription factors. Front Plant Sci 2017;7:2045. https://doi.org/10.3389/fpls.2016.02045. 10.3389/fpls.2016.02045 PMC5222873 28119722

[58] Leyva-González, M. A., Ibarra-Laclette, E., Cruz-Ramírez, A., & Herrera-Estrella, L. Functional and transcriptome analysis reveals an acclimatization strategy for abiotic stress tolerance mediated by Arabidopsis NF-YA family members. PLoS One 2012; 7(10), e48138. https://doi.org/10.1371/journal.pone.0048138. 10.1371/journal.pone.0048138 PMC3485258 23118940

[59] Li, Y. J., Fang, Y., Fu, Y. R., Huang, J. G., Wu, C. A., & Zheng, C. C. NFYA1 is involved in regulation of postgermination growth arrest under salt stress in Arabidopsis. PLoS One 2013; 8(4), e61289. https://doi.org/10.1371/journal.pone.0061289. 10.1371/journal.pone.0061289 PMC3634844 23637805

[60] Munns, Rana, and Mark Tester. “Mechanisms of salinity tolerance.” Annu Rev Plant Biol 2008; 59: 651–681. https://doi.org/10.1146/annurev-arplant-050718-100005. 10.1146/annurev.arplant.59.032607.092911 18444910

[61] Parihar, P., Singh, S., Singh, R., Singh, V. P., & Prasad, S. M. Effect of salinity stress on plants and its tolerance strategies: a review. Environ Sci Pollut Res Int 2015; 22(6), 4056–4075. https://doi.org/10.1007/s11356-014-3739-1. 10.1007/s11356-014-3739-1 25398215

[62] Uddin, M. N., Hossain, M. A., and Burritt, D. “Salinity and drought stress: similarities and differences in oxidative responses and cellular redox regulation,” in Water Stress and Crop Plants: A Sustainable Approach, ed. P. Ahmad (Hoboken, NJ: Wiley). 2016; 86–101. https://doi.org/10.1002/9781119054450.ch7.

[63] Kumari, A., Das, P., Parida, A. K., & Agarwal, P. K. Proteomics, metabolomics, and ionomics perspectives of salinity tolerance in halophytes. Front Plant Sci 2015; 6,537. https://doi.org/10.3389/fpls.2015.00537. 10.3389/fpls.2015.00537 PMC4518276 26284080

[64] Corrêa TR, Motoike SY, Coser SM, DA Silveira G, De Resende MDV, Chia GS (2015). estimation of genetic parameters for *in vitro* oil palm characteristics (*Elaeis guineensis* Jacq.) and selection of genotypes for cloning capacity and oil yield. Ind Crop Prod.

[65] OmicsBox – Bioinformatics Made Easy, BioBam Bioinformatics, March 3, 2019. https://www.biobam.com/omicsbox

[66] Dobin, A., Davis, C. A., Schlesinger, F., Drenkow, J., Zaleski, C., Jha, S., Batut, P., Chaisson, M., & Gingeras, T. R. STAR: ultrafast universal RNA-seq aligner. Bioinformatics 2013; 29(1), 15–21. https://doi.org/10.1093/bioinformatics/bts635. 10.1093/bioinformatics/bts635 PMC3530905 23104886

[67] Andrews S. FastQC: a quality control tool for high Thoughput sequence 2010. Data. Retrieved 2018, from https://www.bioinformatics.babraham.ac.uk/projects/fastqc/

[68] Bolger, A. M., Lohse, M., & Usadel, B. Trimmomatic: a flexible trimmer for Illumina sequence data. Bioinformatics 2014; 30(15), 2114–2120. https://doi.org/10.1093/bioinformatics/btu170. 10.1093/bioinformatics/btu170 PMC4103590 24695404

[69] Anders, S., Pyl, P. T., & Huber, W. HTSeq--a Python framework to work with high-throughput sequencing data. Bioinformatics 2015; 31(2), 166–169. https://doi.org/10.1093/bioinformatics/btu638. 10.1093/bioinformatics/btu638 PMC4287950 25260700

[70] Robinson, M. D., McCarthy, D. J., & Smyth, G. K. EdgeR: a Bioconductor package for differential expression analysis of digital gene expression data. Bioinformatics 2010; *26*(1), 139–140. https://doi.org/10.1093/bioinformatics/btp616. 10.1093/bioinformatics/btp616 PMC2796818 19910308

[71] Martin, Marcel. Cutadapt removes adapter sequences from high-throughput sequencing reads. EMBnet.journal, [S.l.], v. 17, n. 1, p. pp. 10–12, may 2011. ISSN 2226–6089. Available at: <http://journal.embnet.org/index.php/embnetjournal/article/view/200>. Date accessed: 15 july 2020. doi:https://doi.org/10.14806/ej.17.1.200

[72] Langmead, B., Trapnell, C., Pop, M., et al. Ultrafast and memory-efficient alignment of short DNA sequences to the human genome. Genome Biol 2009 10, R25. https://doi.org/10.1186/gb-2009-10-3-r25. 10.1186/gb-2009-10-3-r25 PMC2690996 19261174

[73] Axtell, M. J., & Meyers, B. C. Revisiting criteria for plant MicroRNA annotation in the era of big data. Plant Cell 2018, 30(2), 272–284. https://doi.org/10.1105/tpc.17.00851. 10.1105/tpc.17.00851 PMC5868703 29343505

[74] Tarazona, S., Furió-Tarí, P., Turrà, D., Pietro, A. D., Nueda, M. J., Ferrer, A., & Conesa, A. Data quality aware analysis of differential expression in RNA-seq with NOISeq R/bioc package. Nucleic Acids Res 2015, 43(21), e140. https://doi.org/10.1093/nar/gkv711. 10.1093/nar/gkv711 PMC4666377 26184878

[75] Blum, M., Chang, H, et al. The InterPro protein families and domains database: 20 years on. Nucleic Acids Research, Nov 2020, https://doi.org/10.1093/nar/gkaa977. 10.1093/nar/gkaa977 PMC7778928 33156333

